# Contrast-enhanced ultrasound with VEGFR2-targeted microbubbles for monitoring combined anti-PD-L1/anti-CTLA-4 immunotherapy effects in a murine melanoma model with immunohistochemical validation

**DOI:** 10.1371/journal.pone.0326675

**Published:** 2025-07-01

**Authors:** Felix L. Herr, Melissa J. Antons, Larissa V. Blume, Heidrun Hirner-Eppeneder, Sandra Kloiber-Langhorst, Amra Cimic, Jennifer Stueckl, Isabelle Tardy, Tanja Burkard, Jens Ricke, Wolfgang G. Kunz, Dirk-Andre Clevert, Maurice M. Heimer, Clemens C. Cyran

**Affiliations:** 1 Department of Radiology, LMU University Hospital Munich, Munich, Germany; 2 Bracco Suisse South Australia, Geneva, Switzerland; European Institute of Oncology, ITALY

## Abstract

**Background:**

Immune checkpoint inhibitors (ICIs) have emerged as a highly effective treatment option for patients with metastatic melanoma. As not all patients respond to ICI immunotherapy, imaging biomarkers are required to accurately monitor early response to therapy. Therefore, the aim of this study was to evaluate contrast-enhanced ultrasound (CEUS) with VEGFR2-targeted microbubbles for monitoring the effects of combined anti-PD-L1/anti-CTLA-4 immunotherapy in a murine melanoma model.

**Methods:**

Murine melanoma allografts (B16-F10) were implanted subcutaneously in *n* = 10 therapy and *n *= 10 control female C57BL/6 mice. CEUS with VEGFR2-targeted microbubbles was performed on day 7 and 12. The therapy group received 3 intraperitoneal injections on days 7, 9, 11 of combined anti-PD-L1/anti-CTLA-4 immunotherapy, the control group received a placebo. CEUS assessed tumour perfusion during an early vascular phase (wash-in area under the curve = WiAUC) and VEGFR2-specific binding during a late molecular phase (signal intensity at 8 minutes (SI_8min_) and 10 minutes (SI_10min_)). For pathophysiological validation immunohistochemistry was performed.

**Results:**

At follow-up, the CEUS perfusion parameter WiAUC demonstrated a significantly higher decrease in the therapy than in the control group (p = 0.021). At follow-up, the signal enhancement in the late phase was significantly lower in the therapy than in the control group (SI_8min_ p = 0.003; SI_10min_ p = 0.002). Immunohistochemistry revealed significantly more apoptotic tumour cells (p = 0.001), more tumour infiltrating lymphocytes (p = 0.049), lower tumour cell proliferation (p = 0.001), lower microvascular density (p = 0.003) and lower VEGFR2 expression (p = 0.003) in the therapy than in the control group.

**Conclusions:**

CEUS with VEGFR2-targeted microbubbles allowed for monitoring early treatment effects of a combined anti-PD-L1/anti-CTLA-4 immunotherapy on melanoma allografts with significantly lower tumour perfusion and significantly lower binding of VEGFR2-targeted microbubbles in the therapy than in the control group.

## Introduction

In recent years, immune checkpoint inhibitors (ICIs) have emerged as a highly efficacious treatment option for patients with metastatic tumours, and have also been approved for use in melanoma [1]. ICIs restore anti-tumour immune activity by blocking inhibitory pathways that normally dampen T-cell responses [2]. Cytotoxic T-lymphocyte-associated protein 4 (CTLA-4) primarily regulates T-cell activation at the priming phase within lymph nodes by outcompeting the co-stimulatory receptor CD28 for binding to CD80/CD86 on antigen-presenting cells [2]. In contrast, the programmed death 1 (PD-1)/programmed death-ligand 1 (PD-L1) axis suppresses T-cell activity in peripheral tissues, including the tumour microenvironment, by inhibiting effector T-cell function upon ligand engagement [2]. Antibodies targeting CTLA-4 or PD-1/PD-L1 can thereby reinvigorate T-cell responses and promote tumour rejection. These demonstrated considerable clinical success in several malignancies, particularly advanced melanoma. However, clinical response to ICI therapy remains highly variable. For instance, long-term data from the CheckMate 067 trial demonstrated that only about 49% of patients with advanced melanoma were still alive at 6.5 years under combination immunotherapy, and a substantial subset of patients (~40–60%) fail to achieve durable clinical benefit despite treatment [3]. Given that not all patients respond to immunotherapy with ICI and its treatment is associated with adverse effects and the high costs of therapy, imaging biomarkers need to be employed for accurate monitoring of early therapy response [4]. Molecular imaging tools may help address this gap by enabling dynamic assessment of tumour microvascular changes that precede volumetric or clinical response.

Vascular endothelial growth factor (VEGF) is a principal regulator of angiogenesis [5]. VEGF and its receptor VEGFR2 are increasingly expressed in solid tumours, such as melanoma, and play an important role in tumours angiogenesis, proliferation and formation of metastases [6–9]. Furthermore, VEGFR2 is associated with the development of resistance to anti-cancer drugs [10]. A correlation between tumour immune microenvironment and VEGF(R-2) has already been described: The activation of VEGF(R-2) is associated with the development of an immunosuppressive microenvironment, by inducing the aggregation of immature dendritic cells, bone marrow-derived suppressor cells and regulatory T cells, and inhibiting T lymphocyte migration [11–13]. Moreover, VEGF(R-2) has been demonstrated to enhance PD-1 expression [11,14]. Studies have shown that VEGFR2 mutations have been associated with improved clinical outcomes for tumours under ICI [15]. Additionally, VEGFR2 expression level can serve as an important indicator of how well patients will respond to therapies that block PD-1 and PD-L1 [16]. Further, immunotherapy can reduce the expression of VEGF, which helps counteract its immunosuppressive effects [17,18].

Contrast-enhanced ultrasound (CEUS) utilising microbubbles (MB) that bind specifically to VEGFR2 enables the characterisation of functional and molecular parameters [19,20]. The MBs are exclusively distributed intravascularly [21]. Due to their size (1–5 μm), microbubbles are physically restricted to the intravascular space and cannot extravasate. BR55 microbubbles bind specifically to VEGFR2 expressed on the luminal surface of endothelial cells, allowing for intravascular molecular imaging of tumour angiogenesis [21,22]. Following intravenous injection, the perfusion of tumours can be quantitatively evaluated in an early phase by assessing various parameters of tissue microcirculation (perfusion). In a subsequent late phase, targeted MBs bound to tumour vascular endothelial cells, which overexpress VEGFR2, permit the non-invasive visualisation of VEGFR2 expression [23,24]. Multiparametric CEUS with VEGFR2-targeted MB has demonstrated to be a highly sensitive tool for monitoring therapy effects in a range of tumour entities: In a mouse model of hepatocellular carcinoma, early therapeutic effects of the tyrosine kinase inhibitor sorafenib were analysed [25]. Eschbach et al. has already used CEUS to analyse morphology, perfusion and VEGFR2-specific binding in colorectal adenocarcinoma in rats [26]. Consequently, VEGFR2-targeted CEUS may provide functional and molecular imaging biomarkers for the assessment of tumour angiogenesis and early therapy response in murine melanoma allografts under immunotherapy.

Therefore, we hypothesised that CEUS with VEGFR2-targeted MB enables monitoring of early immunotherapy effects in a murine melanoma allograft tumour model. We aimed to investigate whether the functional and the molecular CEUS parameters have potential as non-invasive in vivo imaging biomarkers of early therapy response with time point-matched multiparametric immunohistochemical validation.

## Materials and methods

### Animal model and experimental protocol

All animal experiments were conducted in accordance with the guidelines for the use of living animals in scientific studies and the animal study was officially approved by the Committee for Animal Research of the Government of Upper Bavaria (ROB-55.2–2532.Vet_02-19-32). The authors have ensured compliance with the ARRIVE guidelines. All efforts were made to minimize suffering. Female C57BL/6 mice (mean weight ± 20 g, aged 10–12 weeks at the time of inoculation, Charles River, Sulzfeld, Germany) were housed in groups of four per cage in a temperature- and humidity-controlled room with a 12 h light/dark cycle and provided with food and water. In accordance with the approved animal study proposal, animals were excluded from the study if they exhibited any of the following symptoms: a loss of body weight equal to or greater than 19% of the value observed prior to inoculation; a tumour size exceeding 1.5 cm; exulceration; infections or bleeding from the tumour area; bloody diarrhoea; apathy; ascites; or acneiform dermatitis. Animals were monitored daily for signs of distress. Humane endpoints were predefined and animals showing signs of suffering were euthanized immediately. All efforts were made to minimize pain and distress. Analgesia was administered when deemed necessary in accordance with the approved protocol to alleviate potential pain and distress.

The murine melanoma cell line B16-F10 (ATCC CRL-6475) was cultured and expanded under standard conditions. After a one-week assimilation period, the melanoma cells (B16-F10; 3 x 10^5^ cells) were resuspended in a 1:1 mixture of Matrigel (BD Biosciences, San Jose, CA, USA) and phosphate-buffered saline (PBS, pH 7.4). The tumour cells were injected subcutaneously into the left abdominal flank of C57BL/6 mice (*n* = 20) under inhalative isoflurane anaesthesia, with 2.0 vol % isoflurane and 1.5 L/min oxygen. Anaesthesia was maintained throughout imaging sessions using a continuous flow of the same gas mixture. Analgesia was administered post-interventionally when deemed necessary to alleviate pain, in accordance with the approved study protocol. Once tumour diameter reached a maximum of 0.5 cm (typically on day 7 post-inoculation), animals were randomly assigned to either the therapy (*n* = 10) or the control group (*n* = 10) and baseline CEUS was conducted on day 7 post-inoculation. The therapy group received a total of three intraperitoneal injections of anti-PD-L1 (inVivoPlus anti-mouse PD-L1 (B7-H1), BioXCell, #PB0101) and anti-CTLA-4 (InVivoPlus anit-mouse CTLA-4 (CD152), BioXCell, #PB0131) antibodies (20 µg/kg) on days 7, 9 and 11 after inoculation. The control group received volume equivalent placebo. For imaging, anaesthesia was maintained via continuous inhalation of 2.0 vol % isoflurane and 1.5 L/min oxygen and were placed on a heating pad. A multiparametric CEUS protocol was conducted on day 7 (baseline) and on day 12 (follow-up) following inoculation. Mice were euthanized by cervical dislocation under deep isoflurane anaesthesia in accordance with institutional and governmental animal welfare regulations. This method was selected to ensure rapid and humane sacrifice following anaesthetic-induced loss of consciousness. For histological analysis, tumours were explanted at baseline (day 7) and follow-up (day 12) from an independent immunohistochemical cohort, separate from the CEUS-imaged animals. Explanted tissue was fixed in formalin and preserved by cryoconservation for subsequent multiparametric immunohistochemical evaluation.

### Study design overview

A total of 36 mice were used in this study. Twenty mice were allocated to the CEUS imaging cohort and were randomly divided into a therapy group (*n* = 10) and a control group (*n* = 10) after tumour establishment. The therapy group received intraperitoneal injections of anti-PD-L1 and anti-CTLA-4 antibodies on days 7, 9, and 11 post-inoculation. CEUS imaging was performed on day 7 (baseline) and day 12 (follow-up).

**Fig 1:**
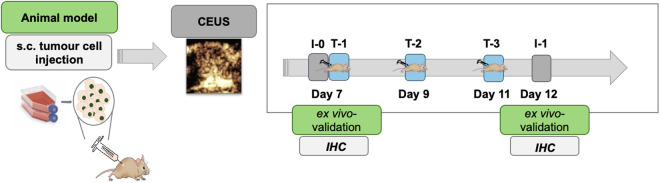
Experimental design. Experimental timeline showing therapy days (7, 9, 11), CEUS imaging time points (day 7 and 12), and immunohistochemical analysis time points (day 7 and 12). The control group received a placebo, while the therapy group received combined anti-PD-L1/anti-CTLA-4 immunotherapy on days 7, 9 and 11 after inoculation. CEUS imaging was performed in one cohort of animals on days 7 and 12. Immunohistochemical analysis was conducted in a separate, dedicated cohort of animals with tumour explantation at day 7 or day 12.

For time-matched histological validation, an additional, independent cohort of 16 mice (*n* = 8 therapy, *n* = 8 control) was used exclusively for immunohistochemical analysis. In this immunohistochemical cohort, tumours were explanted at day 7 (baseline) or day 12 (follow-up), respectively. The overall study timeline is illustrated in Fig 1.

### Microbubbles

BR55 (Bracco Suisse SA, Geneva, Switzerland) is an ultrasound molecularly targeted lipid-shelled microbubble that targets VEGFR2. The lipopeptide that binds to VEGFR2 is created by conjugating a heterodimer peptide to the amino group of DSPE-PEG2000-NH2, which is then incorporated into the phospholipid-based MB formulation. The final product, BR55, is provided as a lyophilized powder in a septum-sealed vial, with the gas phase consisting of a mixture of perfluorobutane and nitrogen [22,27,28]. Prior to utilisation, BR55 must be reconstituted by the injection of 2 ml of a 5% glucose solution into the vial via the septum. Once dissolved, the resulting MB suspension is ready for utilisation. The mean diameter of the BR55 MBs is 1.5 μm, with a concentration of approximately 2 x 10⁹ MB per mL. Each MB contains approximately 4 x 10⁵ lipopeptide molecules [22,29]. The circulation time of BR55 is approximately 4 minutes [24]. The administration of BR55 to each animal was conducted via a standardised manual intravenous injection through a tail vein catheter, with a dose of 200 μL administered.

### CEUS imaging

CEUS imaging was conducted using a clinical ultrasound system (Philips Epiq 7, Seattle, WA) with a conventional 18L4 linear transducer (transmit frequency 9 MHz, dynamic range 55 dB, depth 25 mm), linear time-gain compensation, and an acoustic focus positioned at the largest tumour cross-section, with the contrast mode activated using the system’s image setting [22]. The anaesthetised mice were positioned on their sides, and ultrasound coupling gel was applied to the shaved skin. An initial B-mode scan was conducted to visualise the subcutaneous tumour expansion. Video acquisition commenced immediately prior to the administration of BR55, continued for the initial minute, and then resumed at two-minute intervals for a period of up to ten minutes post-injection (at a frame rate of 16 Hz). This enabled the measurement of MB wash-in and wash-out in the tumour. The binding of the VEGFR2-specific agent was evaluated at 8 and 10 minutes after the injection of BR55, when the circulating MB had largely cleared from the bloodstream, while the tumour tissue remained highlighted, indicating the specific accumulation of targeted MB on the VEGFR2-expressing endothelium.

### Data post processing

**Fig 2:**
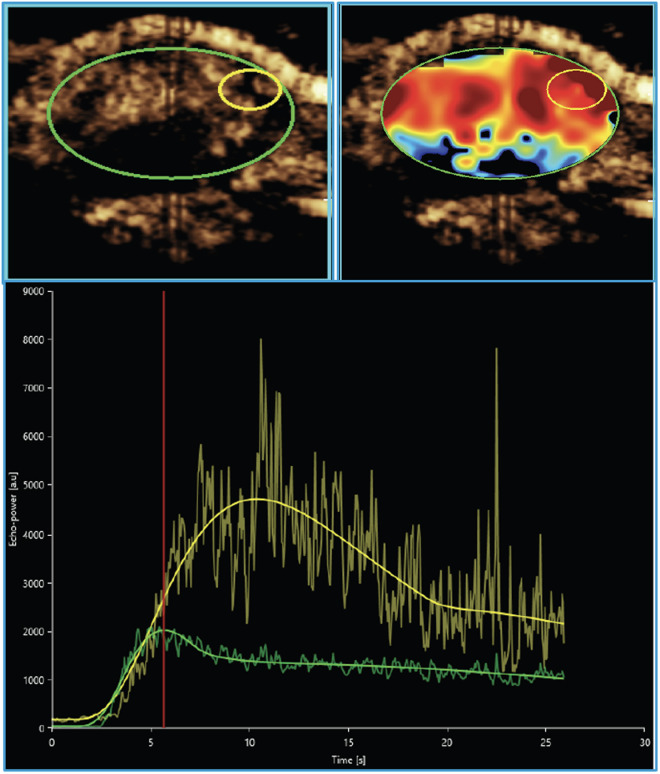
ROI selection. Representative CEUS images acquired seven days after tumour inoculation (baseline) showing tumour (green) and a blood volume parameter map with a ROI in a hypervascular vital tumour site of the outer rim (yellow) are shown. The corresponding signal-intensity-versus-time curves are displayed below.

The CEUS data were processed using dedicated software on an external workstation with Vuebox® (Version v7.5.0.7051 - 64-bit version – Bracco Suisse SA, Geneva, Switzerland). A region of interest (ROI) was delineated within a highly perfused area of the tumour’s vital outer rim without necrotic areas. The software quantifies contrast enhancement in the ROI, presenting results as relative echo-power values that correspond to MB concentration, measured in arbitrary units (a.u.) [22,23,25] (Fig 2). The key parameters analysed included the wash-in area under the curve (WiAUC) during the early vascular phase, serves as a surrogate for tumour blood flow and volume [30,31], as well as signal intensity measured at 8 minutes (SI_8min_) and 10 minutes (SI_10min_) post-injection, which serve as surrogate indicators of VEGFR2-specific MB binding in the late molecular phase. WiAUC has been established as a surrogate marker of tumour perfusion and correlates with microvascular density and blood volume, as demonstrated in previous CEUS studies [22,31]. In addition, the size of the tumour was measured in two dimensions.

### Immunohistochemistry

#### CD31.

First deparaffinization, rehydration and antigen retrieval (SignalStain^®^ EDTA-Unmasking Solution, Cell signaling Technology, Leiden, Netherlands) was performed. For immunohistochemical assessment of tumour microvascular density, non-specific binding sides were blocked in 5% Donkey-Serum in TBS-Tween20. Subsequently tumours slices were incubated with a polyclonal rabbit anti-CD31 primary antibody (1:50; Abcam ab28364, Cambridge, UK) overnight. Tissue samples underwent further processing utilising secondary antibodies conjugated with Alexa Fluor 488 (1:200; ab150073, Abcam Limited, Cambridge, UK), to facilitate fluorescence detection. Counterstaining was conducted using DAPI (Carl Roth, Karlsruhe, Germany) and the slides were covered with Fluoromount-G (ThermoFisher Scientific, Waltham, Massachusetts, USA). The number of tumour microvessels was quantified as the mean of the endothelial cells in 10 random fields at 200x magnification, as previously described [32].

#### CD8 antigen staining.

The tissue slides were initially deparaffinised and rehydrated, followed by permeabilization with a solution of 1x PBS (Gibco, Carlsbad, CA) and 0.25% Triton-X-100 (Merck KGaA, Darmstadt, Germany). Subsequently, antigen retrieval was conducted by boiling the tissue slides in a 10 mM Tris-EDTA buffer for CD8 (Life Technologies, Carlsbad, CA). Following the retrieval of antigens, thorough washing with 1x PBS was conducted to ensure the removal of residual buffers. Non-specific binding sites were blocked with a solution containing bovine serum albumin (BSA) and primary antibodies against CD8 (1:50; ab217344, Abcam Limited, Cambridge, UK) were applied. Following an overnight incubation period and additional washing steps, secondary antibodies conjugated with Alexa Fluor 488 (1:200; ab150073, Abcam Limited, Cambridge, UK) were applied to facilitate fluorescence detection. Counterstaining with DAPI enabled the visualisation of nuclei. Finally, slides were mounted using Fluoromount-G, ensuring the preservation and optimal visualisation of the stained specimens. Results were quantified as the mean percentage of CD8-positive T-cells in 10 randomly selected fields at 200x magnification.

#### Ki-67 antigen staining.

A Ki-67-specific recombinant rabbit monoclonal antibody (1:50; SP6, Thermo Fisher MA5–14520, Thermo Fisher) was employed for the purpose of quantifying tumour cell proliferation. The tissue was de-masked in Universal antigen retrival reagent (ab208572, Abcam Limited, Cambridge, UK) using microwave irradiation at 600 W. Following a wash in distilled water and TBS-Tween (0.05%), secondary antibodies conjugated with Alexa Fluor 488 (1:200; ab150073, Abcam Limited, Cambridge, UK) were applied to facilitate fluorescence detection. Subsequently, counterstaining was conducted using DAPI (Carl Roth, Karlsruhe, Germany) and slides were covered with Fluoromount-G (ThermoFisher Scientific, Waltham, Massachusetts, USA). Results were quantified as the mean percentage of proliferating cells in 10 random fields at 200x magnification.

#### Terminal desoxynucleotidyl transferase dUTP nick-end labelling (TUNEL).

The initial step involved the deparaffinisation and rehydration of the tissue slides, which were then permeabilised using a solution comprising Triton X-100 (Merck KGaA, Darmstadt, Germany) and sodium citrate (Sigma-Aldrich, Steinheim, Germany). Subsequently, antigen retrival reagent (ab208572, Abcam Limited, Cambridge, UK) was conducted through microwave treatment. The staining was conducted by combining solutions from the provided kit, with meticulous attention to maintaining darkness and temperature control. Following comprehensive washing steps, slides were further stained with DAPI to visualise nuclei. Ultimately, slides were mounted using Fluoromount-G (ThermoFisher Scientific, Waltham, Massachusetts, USA) ensuring the preservation of the stained samples for microscopic examination. Results were quantified as the mean percentage of apoptotic cells in 10 random fields at 200x magnification.

#### VEGFR2.

The expression of VEGFR2 was analysed using a VEGFR2-specific monoclonal rabbit antibody (1:50; #2479, Cell Signalling, Cambridge, UK). The antibody used specifically detects endogenous VEGFR2 in both human and murine endothelial cells. Therefore, the VEGFR2 expression observed reflects murine endothelial VEGFR2 in our allograft model. Following standard procedures, the tissue samples were dewaxed, rehydrated and subsequently demasked in target-retrieval solution (SignalStain EDTA Unmasking Solution, Cell Signalling, Cambridge, UK) using microwave irradiation at 600 W. After an overnight incubation period with the primary antibody, the tissue samples were further processed using secondary antibodies conjugated with Alexa Fluor 488 (1:200; ab150073, Abcam Limited, Cambridge, UK) and counterstaining was conducted using DAPI (Carl Roth, Karlsruhe, Germany). The number of tumour vessels stained positively for VEGFR2 was quantified at a magnification of 200x, with the analysis conducted on 10 random high-power fields.

### Statistical analysis

Continuous variables are presented as means with standard deviations (mean ± SD). For intergroup comparisons between treatment and control group the Mann-Whitney U-test was employed. A Wilcoxon signed-rank test was used for intragroup comparisons of CEUS parameters between baseline (day 7) and follow-up (day 12). P-values < 0.05 were considered statistically significant. All statistical analyses were performed using GraphPad Prism (Graphpad Software, Inc., Boston, MA) and Microsoft Excel (Microsoft Corporation, Redmond, WA).

## Results

The experimental protocol, including imaging with CEUS, was successfully completed in *n *= 16 animals; in *n *= 3 animals the ultrasound could not be accomplished due to technical issues, *n *= 1 animal of the therapy group had to be excluded due to anaesthesia complications.

### Tumour size

Tumour size was assessed at baseline (7 days after inoculation) and follow-up (12 days after inoculation). For calliper measurements in two dimensions there were no significant differences in mean tumour sizes between the therapy and the control group at baseline (29.9 ± 12.7 mm^2^ vs. 32.1 ± 17.3 mm^2^; p = 0.909) and follow-up (104.2 ± 35.3 mm^2^ vs. 123.0 ± 66.1 mm^2^; p = 0.733). There were significant changes in tumour size between baseline and follow-up in the therapy (29.9 ± 12.7 mm^2^ vs. 104.2 ± 35.3 mm^2^; p = 0.006) and in the control group (32.1 ± 17.3 mm^2^ vs. 123.0 ± 66.1 mm^2^; p = 0.002, S1 Table). For CEUS measurements in two dimensions there were no significant differences in mean tumour sizes between the therapy and the control group at baseline (21.3 ± 13.2 mm^2^ vs. 17.4 ± 19.8 mm^2^; p = 0.173) and follow-up (60.2 ± 28.1 mm^2^ vs. 63.2 ± 48.6 mm^2^; p = 1). There were significant increases in tumour size between baseline and follow-up in the therapy (21.3 ± 13.2 mm^2^ vs. 60.2 ± 28.1 mm^2^; p = 0.004) and in the control group (17.4 ± 19.8 mm^2^ vs. 63.2 ± 48.6 mm^2^; p = 0.002, S2 Table). Tumour diameters increased from baseline to follow-up in both groups, with no statistically significant difference observed. These measurements served as morphological reference values in parallel to functional imaging analysis.

### CEUS

**Fig 3:**
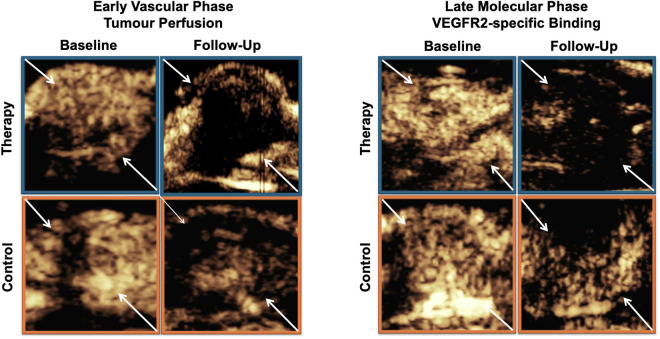
Representative CEUS images with BR55 of mice with a subcutaneous tumour allograft (arrows) under treatment. Therapy (blue) and control group (orange) at baseline and follow-up. Arrows indicating tumour allografts. Left side: early vascular phase as a functional imaging biomarker; right side: late phase demonstrating VEGFR2-specific binding as a molecular imaging biomarker, 10 minutes post-contrast injection. At follow-up, it is notable that the number of circulating MB observed in the early vascular phase was significantly lower in the therapy group compared to both the baseline and the control group. Similarly, signal enhancement in the late phase at follow-up was significantly lower in the therapy group compared to both the baseline and the control group.

Using BR55-CEUS, functional parameters of tumour perfusion were assessed during an early vascular phase and VEGFR2-specific binding of the MB during a late molecular phase (Fig 3).

**Fig 4:**
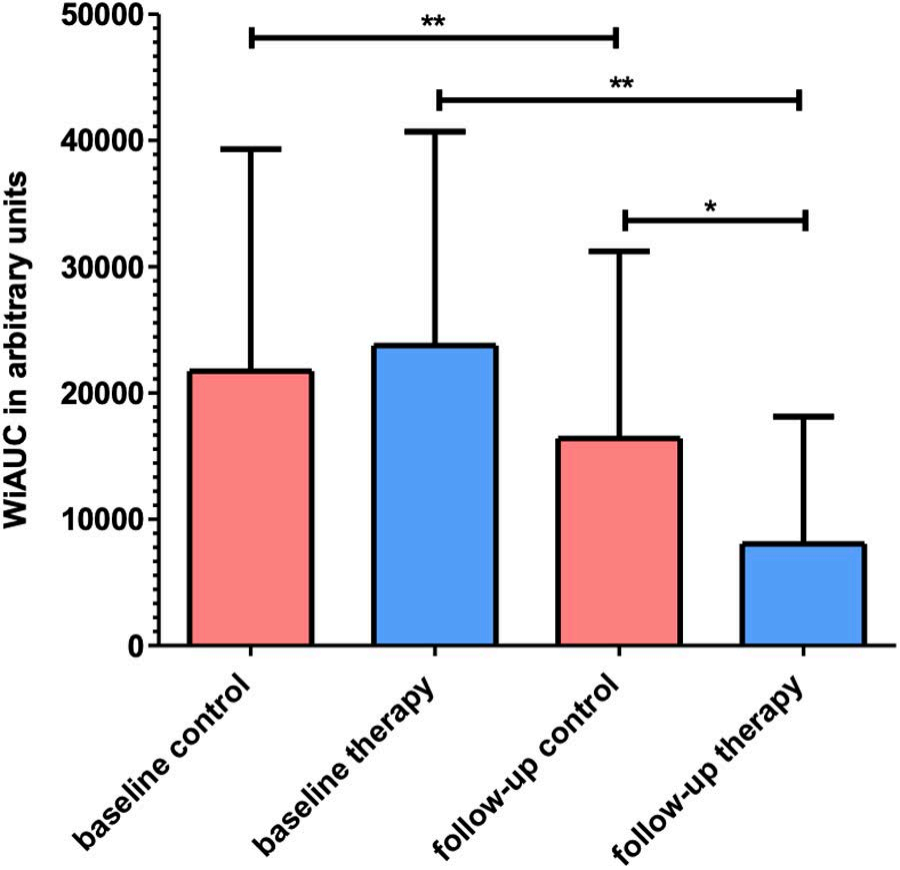
Column charts of WiAUC in therapy and control group at baseline and follow-up. WiAUC in arbitrary units of control (*n* = 8) and therapy group (*n* = 8) at baseline and follow-up. Note the significant difference (** p < 0.02) between the mean values of WiAUC between the therapy (blue) and the control group (red) at follow-up. There is also a significant (** p < 0.02) decline of WiAUC in therapy group between baseline and follow-up. Significantly (* p < 0.05) lower WiAUC values in the therapy compared to the control group at follow-up.

In the therapy group, a significant decrease of tumour perfusion (WiAUC) was observed following the one-week treatment course with combined anti-PD-L1/anti-CTLA-4 immunotherapy (23767 ± 16937 a.u. at baseline to 8054 ± 10083 a.u. at follow-up; p = 0.008). Also, in the control group significant changes in WiAUC (from 21744 ± 17585 a.u. at baseline to 16402 ± 14813 a.u. at follow-up; p = 0.008) were noted. Moreover, WiAUC was significantly (p = 0.021) lower in the therapy compared to the control group at follow-up (Fig 4 and S3 Table). The reduction in WiAUC from baseline to follow-up was consistently measurable across the majority of animals in the therapy group. Corresponding changes were recorded in the control group as well, though to a lesser extent.

**Fig 5:**
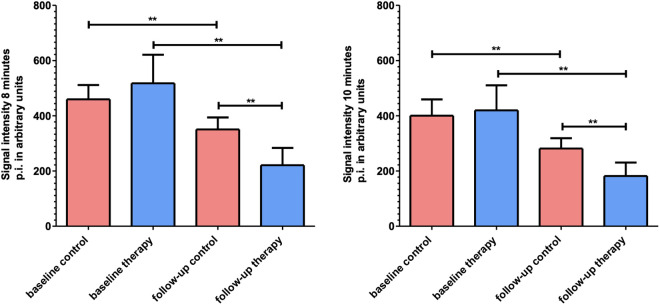
Column charts of SI_8min_ and SI_10min_ in the therapy and control group at follow-up. Signal intensity at 8 minutes (left side) and 10 minutes after injection (right side) in arbitrary units of therapy (*n* = 8) and control (*n* = 8) group at baseline and follow-up. Note the significant difference (** p < 0.02) between the mean values of SI_8min_ and SI_10min_ between the therapy (blue) and the control group (red) at follow-up.

In the late molecular phase, a significant decline in tumour signal intensity between baseline and follow-up was observed in the therapy group (SI_8min_: from 517.7 ± 103.7 to 220.9 ± 62.6 a.u., p = 0.008; SI_10min_: from 419.7 ± 90.4 to 181.9 ± 48.4 a.u., p = 0.008) and in the control group (SI_8min_: from 459.8 ± 51.5 to 350.7 ± 43.3 a.u., p p = 0.008; SI_10min_: from 400.3 ± 59 to 281.5 ± 37.3 a.u., p = 0.008). However, significantly lower SI_8min_ (220.9 ± 62.6 vs. 350.7 ± 43.3 a.u., p = 0.003) and SI_10min_ (181.9 ± 48.4 vs. 281.5 ± 37.3 a.u., p = 0.002) values were detected in the therapy than in the control group at follow-up (Fig 5 and S4 Table). Lower signal intensities at 8 and 10 minutes post-injection were consistently observed in the therapy group at follow-up. The decrease was quantifiable and aligned with the predefined imaging time points.

### Immunohistochemistry

**Fig 6:**
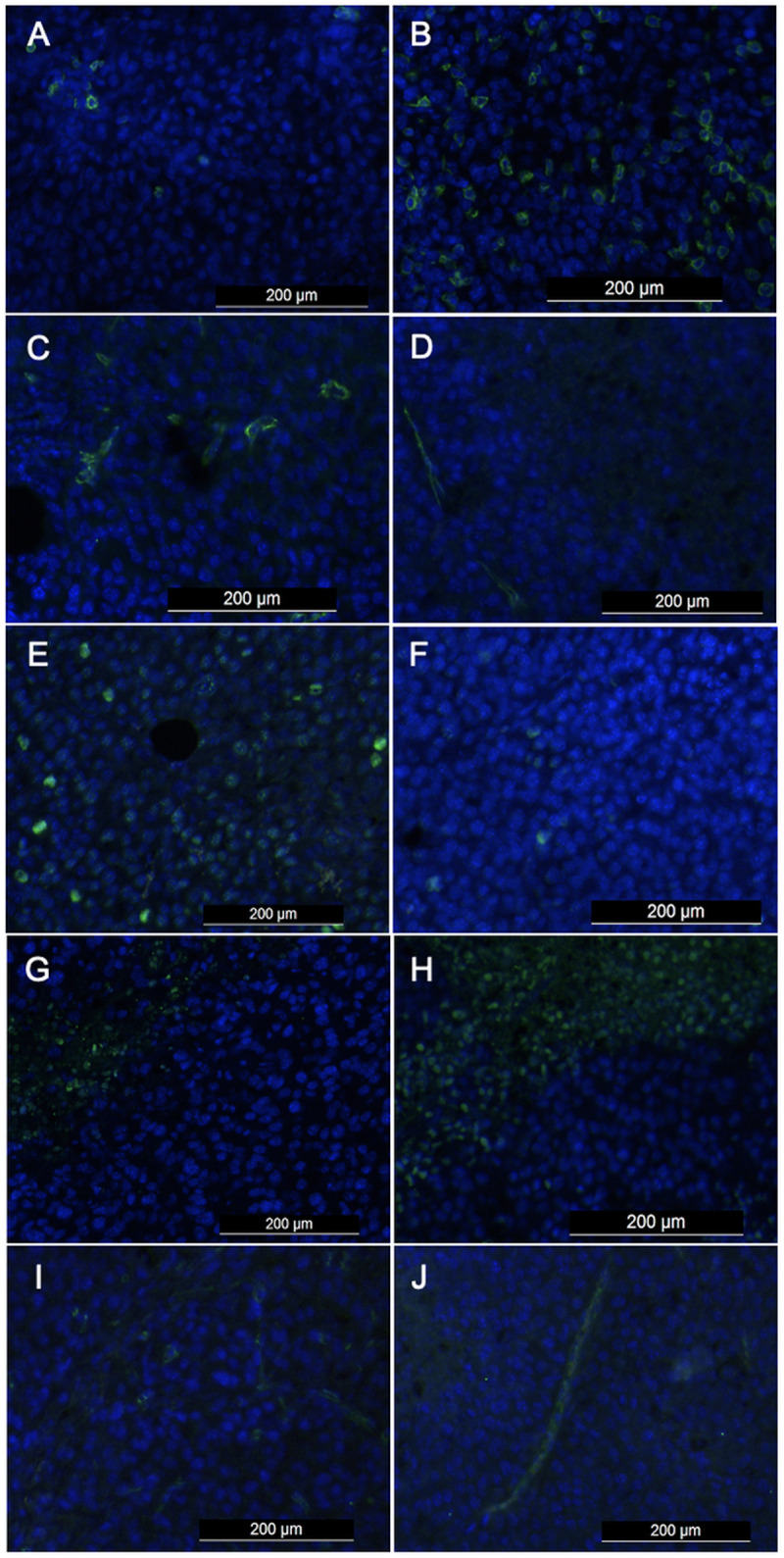
Representative immunohistochemical stainings. CD8 (TILs) (A, B), CD31 (microvascular density) (C, D), Ki-67 (cell proliferation) (E, F), TUNEL (apoptosis) (G, H) and VEGFR2 (I, J) expression in control group (left column) and therapy group (right column). Note that the therapy group exhibited a significantly higher immune response and apoptosis rate, as well as a significantly lower microvascular density, tumour cell proliferation and VEGFR2 expression.

Quantitative ex vivo immunohistochemical analysis was performed at follow-up in both groups (*n* = 8 per group). Histological analysis was conducted in a separate animal cohort to compare cellular parameters at baseline and follow-up, independent of CEUS imaging. Compared to the control group, the tumour samples of the therapy group had a significantly higher percentage of apoptotic cells (TUNEL 52.3 ± 17.2% vs. 17.1 ± 5.6%; p = 0.001) at follow-up. Furthermore, the therapy group showed a significantly higher number of TILs than the control group (CD8 395.4 ± 522.5 vs. 71.9 ± 55.6; p = 0.049) at follow-up. At follow-up, tumour cell proliferation was significantly lower in the therapy group than in the control group (Ki-67 27.2 ± 9.7% vs. 58.7 ± 8.6%; p = 0.001). At follow-up, anti-angiogenic effects of combined anti-PD-L1/anti-CTLA-4 immunotherapy were observed in the investigated melanoma allografts with a significantly lower microvascular density in combined anti-PD-L1/anti-CTLA-4-treated than in non-treated animals (CD31 142.1 ± 59.8 vs. 286.3 ± 88.1; p = 0.003), quantified by CD31 staining. VEGFR2 staining showed a significantly lower VEGFR2 expression in combined anti-PD-L1/anti-CTLA-4-treated than in placebo-treated animals (VEGFR2 72.5 ± 18.6 vs. 119 ± 29.6; p = 0.003, Fig 6). Differences between baseline and follow-up were most pronounced in the therapy group.

## Discussion

Due to severe adverse events and the fact that not all patients respond to ICI, it is necessary to evaluate non-invasive in vivo imaging biomarkers of early therapy response in order to provide effective therapy guidance [33]. Preclinical and clinical studies have investigated the therapy effects, particularly under anti-angiogenic drugs, in melanoma using CEUS [34,35]. However, imaging biomarkers of early therapy response under combined immunotherapy in melanoma are still scarce and require further investigation to ascertain the feasibility of clinical use.

We first assessed tumour size under ICI therapy, as changes in tumour volume are a conventional endpoint in preclinical oncology studies. Tumour size, measured using callipers or CEUS, did not differ significantly between the therapy and control group at follow-up. This indicates that conventional morphological parameters, such as tumour size, are not sensitive enough to detect early physiological or molecular changes in response to immunotherapy [35]. This finding is in accordance with Mellinger [34], who observed no difference in tumour size but a decrease in tumour perfusion under immunotherapy in melanoma xenografts. In contrast to us, Mellinger [34] used one type of antibody (anti-PD1) for checkpoint inhibition, and the therapy was administered twice a week within a 18-day period. These findings further support the notion that immunotherapy does not affect tumour size significantly at early follow-up.

Beyond morphological assessment, we next analysed changes in tumour perfusion using CEUS during the early vascular phase. A significantly higher reduction of WiAUC at follow-up was observed in the therapy compared to the control group. This is in accordance with Eschbach et al. [26] who demonstrated a significant reduction in WiAUC under regorafenib for colorectal carcinoma xenografts. However, their analysis was conducted following a one-week daily treatment of regorafenib. Regorafenib is an multi-tyrosine kinase inhibitor with a distinct mechanism of action that demonstrated anti-angiogenic and anti-proliferative effects in vivo in different experimental tumour models, including breast cancer, renal cell carcinoma and glioblastoma [36]. The significant decrease of the CEUS perfusion parameter WiAUC was consistent with the immunohistochemical CD31 staining, that demonstrated significantly lower microvascular density. This finding is in accordance with Brloznik et al. [37], who were able to correlate a reduction of tumour perfusion in CEUS with tumour histological analyses of CD31 in a murine melanoma model. Differing from our study Brloznik et al. treated the mice with gene electrotransfer and radiotherapy. According to our results, the early vascular CEUS parameter WiAUC indicates to serve as functional perfusion biomarker of early therapy response under combined anti-PD-L1/anti-CTLA-4 immunotherapy in melanoma.

In the late molecular phase of CEUS, we evaluated the effects of ICI therapy on VEGFR2 expression as a marker of anti-angiogenic response. Regarding the evaluation of VEGFR2-targeted microbubbles in the late molecular phase, the treatment group exhibited significantly lower SI_8min_ and SI_10min_ values at follow-up. This is in line with Baetke et al. who demonstrated a significant reduction in the amount of VEGFR2-targeted MB in heterotopic squamous cell carcinoma xenografts in mice undergoing anti-VEGF antibody therapy [24]. In a murine colorectal carcinoma model anti-angiogenic treatment with sunitinib resulted in decreased tumour perfusion and VEGFR2 expression. In contrast to us, they observed treatment effects already 24 hours post-treatment [38]. In accordance with SI_8min_ and SI_10min_ parameters, immunohistochemical measures demonstrated a significant decline in VEGFR2. This is in line with Palmowski et al. [39], who demonstrated, complementary to CEUS, a significantly lower stained area fraction for VEGFR2 in immunohistochemical analysis of treated tumours. Differing from our study, Palmowski et al. investigated squamous cell carcinoma xenografts under a matrix metalloproteinase inhibitor. Notably, we also observed a moderate but significant decrease in WiAUC in the control group between baseline and follow-up. This effect is most likely attributable to the rapid tumour growth in the absence of therapy, leading to the development of necrotic regions with reduced vascularisation and increased interstitial pressure. Similar findings were reported by Eschbach et al. [26], where untreated tumours developed central necrosis, resulting in decreased perfusion measurements despite continued tumour growth. Such necrosis may contribute to a transient reduction in vascular proliferation and VEGFR2 expression, along with decreased BR55 binding [25]. Thus, necrosis may act as a confounding factor, contributing to the decreased differential targeted enhancement observed in treated and untreated melanoma allografts. In our study, ROI placement was deliberately confined to the vital outer tumour rim to minimise this confounding effect; however, some reduction in functional perfusion parameters may still reflect impaired vascular integrity and microcirculation in necrotic or hypoxic tumour regions. The significantly lower number of MB on the tumour endothelial surface following combined immunotherapy most likely reflects the significantly higher decrease of tumour perfusion and VEGFR2 expression compared to the control group.

Although VEGFR2 can also be expressed by tumour cells, CEUS with BR55 specifically targets the luminal VEGFR2 expressed on endothelial cells. Due to their size, microbubbles cannot extravasate or access extravascular tumour cell VEGFR2. This intravascular targeting has been well validated for BR55 in preclinical tumour models [22]. In our experimental setup, the observed reduction of specifically bound MB with VEGFR2-targeted CEUS under anti-PD-L1/anti-CTLA-4 immunotherapy of malignant melanoma may be explained by the pathophysiological connection between VEGF and tumour immunology. The ex vivo validation at follow-up demonstrated a significantly elevated number of TILs and apoptotic cells, as well as a significantly lower number of proliferating cells within the therapy group. These findings indicate the presence of an antitumor immune response under combined anti-PD-L1/anti-CTLA-4 therapy. VEGF via its receptor VEGFR2 contributes to the exhaustion of CD8 + T cells, which is characterised by the expression of negative immune checkpoints, such as PD-1 receptors [40]. In this context, VEGF promotes the expression of checkpoint molecules [11]. Immune checkpoint therapy regulates endothelial cell functions and reverses the immunosuppressive effects of VEGF in metastatic melanoma by decreasing intratumoral VEGF(R2) expression and consecutively activating CD8 + T cells via the IFNγ signalling pathway [17,18,41]. The observed combination of pronounced pro-immunogenic, pro-apoptotic, anti-proliferative and anti-VEGFR2 effects in the immunohistochemistry in our study underscores the effectiveness of the combined immunotherapy and the pathophysiological link between VEGF(R2) and tumour immunology.

The translational relevance of VEGFR2-targeted CEUS is supported by its feasibility and safety in early-phase clinical studies. For example, BR55 has been evaluated in a first-in-human trial in prostate cancer patients [42], where molecular ultrasound imaging correlated with VEGFR2 expression and was well tolerated. Immune checkpoint inhibitors such as anti-PD-L1 and anti-CTLA-4 antibodies are already approved for clinical use in various cancer types, including melanoma and renal cell carcinoma. While immune-related adverse events—particularly in combination regimens—are frequent and potentially severe, they are also well documented and manageable under current clinical guidelines [3,43]. A key translational implication of our findings is that VEGFR2-targeted CEUS may allow early identification of responders and non-responders to ICI therapy. Since immunotherapy response is often delayed and difficult to assess with conventional morphological criteria, molecular CEUS of angiogenic changes could serve as an early, non-invasive surrogate marker of therapeutic efficacy. This could help to personalise treatment strategies, reduce unnecessary exposure to immune-related toxicity, and optimise the timing and selection of subsequent therapies.

The results of our study are limited. Firstly, the investigation was conducted on a single tumour-therapy combination within an allograft model of melanoma. Secondly, the observation period was limited to six days with three doses of therapy. Additionally, CEUS imaging was conducted only until day 12 following tumour inoculation, which was constrained by the dynamics of tumour growth. Double immunofluorescence staining of VEGFR2 and endothelial markers was not feasible in our study due to both antibodies being raised in rabbit, which precluded reliable co-localization. Previous studies have validated the endothelial binding of BR55 under similar conditions [22]. Furthermore, four animals had to be excluded from CEUS analysis due to technical or anaesthesia-related issues. Although these exclusions were not related to treatment effects, they may have limited statistical power and generalisability of the imaging results. Due to rapid tumour progression in the control group, the planned extended follow-up period could not be completed. In accordance with German animal welfare regulations and the approved protocol, animals had to be euthanised once tumour diameter exceeded 1.5 cm. Nonetheless, significant therapy-related changes in perfusion and VEGFR2 expression were already detectable after only five days of ICI treatment, highlighting the early sensitivity of the applied imaging and molecular markers. An extended observation period and longer duration of ICI therapy would likely have resulted in more pronounced effects, including not only further suppression of tumour progression but potentially also measurable tumour regression—outcomes that were beyond the scope of the current study due to ethical constraints.

## Conclusions

This study offers insights into early immunotherapy response in a murine melanoma model, providing time matched correlation of in vivo and ex vivo biomarkers of therapy response. The significant reduction of tumour perfusion and VEGFR2-targeted MB under combined immunotherapy assessed by CEUS were paralleled by significant pro-immunogenic, pro-apoptotic, anti-angiogenic and anti-proliferative effects in immunohistochemistry. Our findings demonstrate that CEUS parameters have potential to serve as non-invasive imaging biomarkers for immunotherapy response assessment in melanoma.

## Supporting information

S1 Table Tumour size in calliper measurements. (DOCX)

S2 Table Tumour size in CEUS measurements. (DOCX)

S3 Table Individual functional CEUS values. (DOCX)

S4 Table Individual molecular CEUS values. (DOCX)
