# Supplementary material for: Contrast-enhanced ultrasound with VEGFR2-targeted microbubbles for monitoring combined anti-PD-L1/anti-CTLA-4 immunotherapy effects in a murine melanoma model with immunohistochemical validation
Source: PLoS One. 2025 Jul 1;20(7):e0326675. doi: 10.1371/journal.pone.0326675 (PMC12212576; doi:10.1371/journal.pone.0326675)
Supplement: S2 Table — (DOCX) [file pone.0326675.s002.docx]

| Animal No. | Tumour Size [mm^2^] | |
| --- | --- | --- |
|  | **Day 0** | **Day 12** |
| THERAPY GROUP | | |
| 1 | 4.0 | 15.0 |
| 2 | 15.0 | 90.0 |
| 3 | 20.0 | 70.0 |
| 4 | 40.0 | 54.0 |
| 5 | 42.0 | 99.0 |
| 6 | 10.0 | 80.0 |
| 7 | 35.0 | 54.0 |
| 8 | 12.0 | 56.0 |
| 9 | 20.0 | n/a |
| 10 | 15.0 | 24.0 |
| Mean | 21.3 | 60.22 |
| SD | 13.17 | 28.13 |
| CONTROL GROUP | | |
| 11 | 3.0 | 48.0 |
| 12 | 2.0 | 56.0 |
| 13 | 8.0 | 117.0 |
| 14 | 8.0 | 96.0 |
| 15 | 60.0 | 182.0 |
| 16 | 30.0 | 180.0 |
| 17 | 9.0 | 143.0 |
| 18 | 42.0 | 252.0 |
| 19 | 6.0 | 60.0 |
| 20 | 6.0 | 96.0 |
| Mean | 17.4 | 123.0 |
| SD | 19.81 | 66.12 |
