# Supplementary material for: Contrast-enhanced ultrasound with VEGFR2-targeted microbubbles for monitoring combined anti-PD-L1/anti-CTLA-4 immunotherapy effects in a murine melanoma model with immunohistochemical validation
Source: PLoS One. 2025 Jul 1;20(7):e0326675. doi: 10.1371/journal.pone.0326675 (PMC12212576; doi:10.1371/journal.pone.0326675)
Supplement: S3 Table — (DOCX) [file pone.0326675.s003.docx]

| Animal No. | WiAUC [a.u.] | |
| --- | --- | --- |
|  | **Day 0** | **Day 12** |
| THERAPY GROUP | | |
| 1 | n/a | n/a |
| 2 | 10582.61 | 1667.95 |
| 3 | 28106.8 | 7375.86 |
| 4 | 12416.58 | 5471.83 |
| 5 | 60080.0 | 32059.07 |
| 6 | 31411.55 | 4135.53 |
| 7 | 12248.78 | 2754.73 |
| 8 | 10510.13 | 1559.15 |
| 9 | n/a | n/a |
| 10 | 24780.45 | 9407.03 |
| Mean | 23767 | 8054 |
| SD | 16937 | 10083 |
| CONTROL GROUP | | |
| 11 | 11059.61 | 10876.04 |
| 12 | 10018.53 | 10017.05 |
| 13 | 26053.72 | 12660.8 |
| 14 | 33148.63 | 21720.51 |
| 15 | 11456.12 | 10216.25 |
| 16 | 59665.41 | 5108963 |
| 17 | 12078.93 | 10139.39 |
| 18 | n/a | n/a |
| 19 | 10470.48 | 4496.72 |
| 20 | n/a | n/a |
| Mean | 21744 | 16402 |
| SD | 17585 | 14813 |
