# Supplementary material for: Contrast-enhanced ultrasound with VEGFR2-targeted microbubbles for monitoring combined anti-PD-L1/anti-CTLA-4 immunotherapy effects in a murine melanoma model with immunohistochemical validation
Source: PLoS One. 2025 Jul 1;20(7):e0326675. doi: 10.1371/journal.pone.0326675 (PMC12212576; doi:10.1371/journal.pone.0326675)
Supplement: S4 Table — (DOCX) [file pone.0326675.s004.docx]

| Animal No. | SI_8min_ [a.u.] | | SI_10min_ [a.u.] | | | |
| --- | --- | --- | --- | --- | --- | --- |
|  | **Day 0** | **Day 12** | **Day 0** | | **Day 12** | |
| THERAPY GROUP | | | | | | |
| 1 | n/a | n/a | | n/a | | n/a |
| 2 | 467.3 | 182.78 | | 349.77 | | 169.4 |
| 3 | 645.24 | 258.62 | | 449.18 | | 227.9 |
| 4 | 673.0 | 347.73 | | 618.4 | | 281.07 |
| 5 | 413.58 | 178.89 | | 395.71 | | 169.78 |
| 6 | 589.43 | 248.3 | | 328.87 | | 154.13 |
| 7 | 464.35 | 180.6 | | 400.37 | | 137.78 |
| 8 | 407.59 | 155.63 | | 372.54 | | 146.17 |
| 9 | n/a | n/a | | n/a | | n/a |
| 10 | 481.3 | 214.43 | | 443.12 | | 169.17 |
| Mean | 517.7 | 220.9 | | 419.7 | | 181.9 |
| SD | 103.7 | 62.57 | | 90.41 | | 48.42 |
| CONTROL GROUP | | | | | | |
| 11 | 440.91 | 355.81 | | 397.3 | | 248.15 |
| 12 | 579.16 | 443.76 | | 510.71 | | 340.5 |
| 13 | 407.07 | 301.55 | | 376.74 | | 276.59 |
| 14 | 456.14 | 367.49 | | 430.39 | | 321.57 |
| 15 | 467.0 | 318.46 | | 368.48 | | 246.0 |
| 16 | 448.06 | 325.49 | | 426.72 | | 281.26 |
| 17 | 430.41 | 347.58 | | 385.64 | | 299.8 |
| 18 | n/a | n/a | | n/a | | n/a |
| 19 | 449.49 | 345.13 | | 306.45 | | 237.77 |
| 20 | n/a | n/a | | n/a | | n/a |
| Mean | 459.8 | 350.7 | | 400.3 | | 281.5 |
| SD | 51.49 | 43.26 | | 59.03 | | 37.28 |
